# Physical activity, body mass index and heart rate variability-based stress and recovery in 16 275 Finnish employees: a cross-sectional study

**DOI:** 10.1186/s12889-016-3391-4

**Published:** 2016-08-02

**Authors:** Tiina Föhr, Julia Pietilä, Elina Helander, Tero Myllymäki, Harri Lindholm, Heikki Rusko, Urho M. Kujala

**Affiliations:** 1 Department of Health Sciences, University of Jyväskylä, P.O. Box 35, FIN-40014 Jyväskylä, Finland; 2 Department of Signal Processing, Tampere University of Technology, P.O. Box 527, FIN-33101 Tampere, Finland; 3 Department of Psychology, University of Jyväskylä, P.O. Box 35, FIN-40014 Jyväskylä, Finland; 4 Finnish Institute of Occupational Health, P.O. Box 40, FIN-00251 Helsinki, Finland; 5 Department of Biology of Physical Activity, University of Jyväskylä, P.O. Box 35, FIN-40014 Jyväskylä, Finland

**Keywords:** Body mass index, Heart rate variability, Physical activity, Physiological stress, Stress, Stress assessment

## Abstract

**Background:**

Physical inactivity, overweight, and work-related stress are major concerns today. Psychological stress causes physiological responses such as reduced heart rate variability (HRV), owing to attenuated parasympathetic and/or increased sympathetic activity in cardiac autonomic control. This study’s purpose was to investigate the relationships between physical activity (PA), body mass index (BMI), and HRV-based stress and recovery on workdays, among Finnish employees.

**Methods:**

The participants in this cross-sectional study were 16 275 individuals (6863 men and 9412 women; age 18–65 years; BMI 18.5–40.0 kg/m^2^). Assessments of stress, recovery and PA were based on HRV data from beat-to-beat R-R interval recording (mainly over 3 days). The validated HRV-derived variables took into account the dynamics and individuality of HRV. Stress percentage (the proportion of stress reactions, workday and working hours), and stress balance (ratio between recovery and stress reactions, sleep) describe the amount of physiological stress and recovery, respectively. Variables describing the intensity (i.e. magnitude of recognized reactions) of physiological stress and recovery were stress index (workday) and recovery index (sleep), respectively. Moderate to vigorous PA was measured and participants divided into the following groups, based on calculated weekly PA: inactive (0 min), low (0 < 150 min), medium (150–300 min), and high (>300 min). BMI was calculated from self-reported weight and height. Linear models were employed in the main analyses.

**Results:**

High PA was associated with lower stress percentages (during workdays and working hours) and stress balance. Higher BMI was associated with higher stress index, and lower stress balance and recovery index. These results were similar for men and women (*P* < 0.001 for all).

**Conclusion:**

Independent of age and sex, high PA was associated with a lower amount of stress on workdays. Additionally, lower BMI was associated with better recovery during sleep, expressed by a greater amount and magnitude of recovery reactions, which suggests that PA in the long term resulting in improved fitness has a positive effect on recovery, even though high PA may disturb recovery during the following night. Obviously, several factors outside of the study could also affect HRV-based stress.

**Electronic supplementary material:**

The online version of this article (doi:10.1186/s12889-016-3391-4) contains supplementary material, which is available to authorized users.

## Background

Physical activity (PA) is known to have positive effects on health [1, 2]. Routine PA reduces stress and enhances psychological wellbeing, which is particularly important for the prevention and management of cardiovascular disease, among other chronic diseases [3]. Regular PA is known to reduce the risk of many adverse health outcomes. Some PA is better than none; however, for most health outcomes, additional benefits are achieved if the amount of PA increases through higher intensity, greater frequency, and/or longer duration. According to the 2008 Physical Activity Guidelines for Americans, most health benefits occur with at least 150 total minutes of moderate intensity or at least 75 min of vigorous intensity aerobic PA per week. However, additional benefits occur with more PA [4, 5]. In addition to the beneficial effects of PA on physical health, these guidelines are also relevant for mental health [6]. Although leisure-time PA has increased among Finnish adults [7], physical inactivity is a major problem and risk for health, in all countries. Furthermore, physical inactivity is associated with being overweight [8] and the current rate of overweight adults worldwide has been described as an epidemic or even a pandemic. This situation is a major public health risk because being overweight is associated with diseases including coronary heart disease, stroke, diabetes and cancer [9].

Together with physical inactivity and overweight, stress at work is a major public health risk. It may even lead to cardiovascular disease [10] without complete recovery [11]. Stress has been shown to reduce participation in leisure-time PA [12, 13]. Furthermore, workplace stress may predict a future increased risk of insufficient PA [14]. Normal weight is associated with good self-reported subjective health [15], including low stress levels [16, 17]. Evidence suggests that psychosocial stress is associated with the development of adiposity [18]. However, according to previous studies, the association between subjective stress and body composition is inconsistent, with evidence both supporting [16, 19] and refuting [20, 21] the idea that stress is associated with adiposity. A recent systematic review reported that the associations of psychosocial factors at work with weight-related outcomes were weak and somewhat inconsistent [22].

Psychological stress causes sympathetic responses in the autonomic nervous system (ANS), such as reduced heart rate variability (HRV) [23]. HRV refers to the variation in intervals between heartbeats and reflects cardiac autonomic modulation. Physiological stress can be defined as an increased body activation level, when sympathetic activity dominates the ANS and parasympathetic activation is low. Stress is associated with reduced HRV, owing to attenuated parasympathetic and/or increased sympathetic activity in cardiac autonomic control. Recovery refers to a reduced body activation level, when parasympathetic activation dominates the ANS over sympathetic activity [24–26]. HRV analysis can be used as a complementary tool to assess general health [27]. HRV analysis during sleep has the potential to explore the sleeping brain, with possible implications for mental health [28]. Previous HRV-studies have mainly used traditional time-domain and frequency-domain measures of HRV, such as root mean square of successive R-R intervals (RMSSD) and the ratio of low frequency power to high frequency power (LF/HF ratio). The traditional measures of HRV represent the average level of the autonomic activity over a period of the time. Cardiac autonomic activity is very dynamic and varies during the day depending on stress, recovery and PA. Therefore, the usability of the traditional measures of HRV is limited in real-life conditions. Additionally, these measures are very individual which further limits their usability in stress assessment and clinical work. However, it is also possible to provide applied heart rate (HR) and HRV-derived stress and recovery variables that take into account the dynamic changes in autonomic activity and individuality of HRV including information that is difficult to obtain from traditional measures of HRV.

The majority of previous studies on the association of PA with stress have used subjective assessment methods or traditional measures of HRV in the assessment of stress. The previous studies support the association of PA with increased HRV [29, 30]. However, accurate and objective methods are needed to reliably assess PA, as well as to assess HRV-based stress and recovery in real-life. By utilizing a method that acknowledges the dynamics and individuality in HRV in real-life, the aim of this study was to investigate the extent to which PA and BMI are associated with HRV-based indicators of stress and recovery on workdays. The study was conducted among 16 275 Finnish employees who had participated in beat-to-beat R-R interval recording as a part of lifestyle counseling between 2007 and 2015. More specifically, accounting for age and sex, we investigated the prevalence of stress and recovery according to the participants’ objectively measured PA level and self-reported body mass index (BMI). Uniqueness of the present study is in the individual and dynamic method used in the assessment of physiological stress and recovery.

## Methods

### Study design and participants

This cross-sectional study investigated the amount and intensity of objective HRV-based stress and recovery on workdays in a real-life sample of 16 275 Finnish employees (6863 men and 9412 women; age 18–65 years; BMI 18.5–40.0 kg/m^2^). The participants nonselectively represent a cross-section of typical Finnish employees including both manual and non-manual labour employees. The majority of the participants were apparently healthy without chronic diseases. The exclusion criteria for participation in the R-R interval recordings included severe cardiac disease, very high blood pressure (≥180/100 mmHg), type 1 or 2 diabetes with autonomic neuropathy, severe neurological disease, fever or other acute disease, and BMI >40 kg/m^2^. These exclusion criteria represented by the analysis software manufacturer are presented in detail previously [31]. The characteristics of the participants are presented in Table 1.

**Table 1:** Characteristics of the participants

Variable	All (*n* = 16275)			Men (*n* = 6863)			Women (*n* = 9412)		
	Mean ± SD	Min	Max	Mean ± SD	Min	Max	Mean ± SD	Min	Max
Age	44.8 ± 9.9	18.0	65.0	44.5 ± 9.9	18.0	65.0	45.0 ± 9.9	18.0	65.0
Body mass index (kg/m^2^)	26.0 ± 4.1	18.5	40.0	26.6 ± 3.5	18.6	40.0	25.5 ± 4.4	18.5	40.0
Self- reported activity class 0–10	4.8 ± 1.8	0.0	10.0	4.9 ± 1.9	0.0	10.0	4.8 ± 1.8	0.0	10.0
Physical activity (mins/week)	186 ± 227	0	2629	246 ± 258	0	2629	142 ± 189	0	1865

### Data collection

**Fig. 1:**
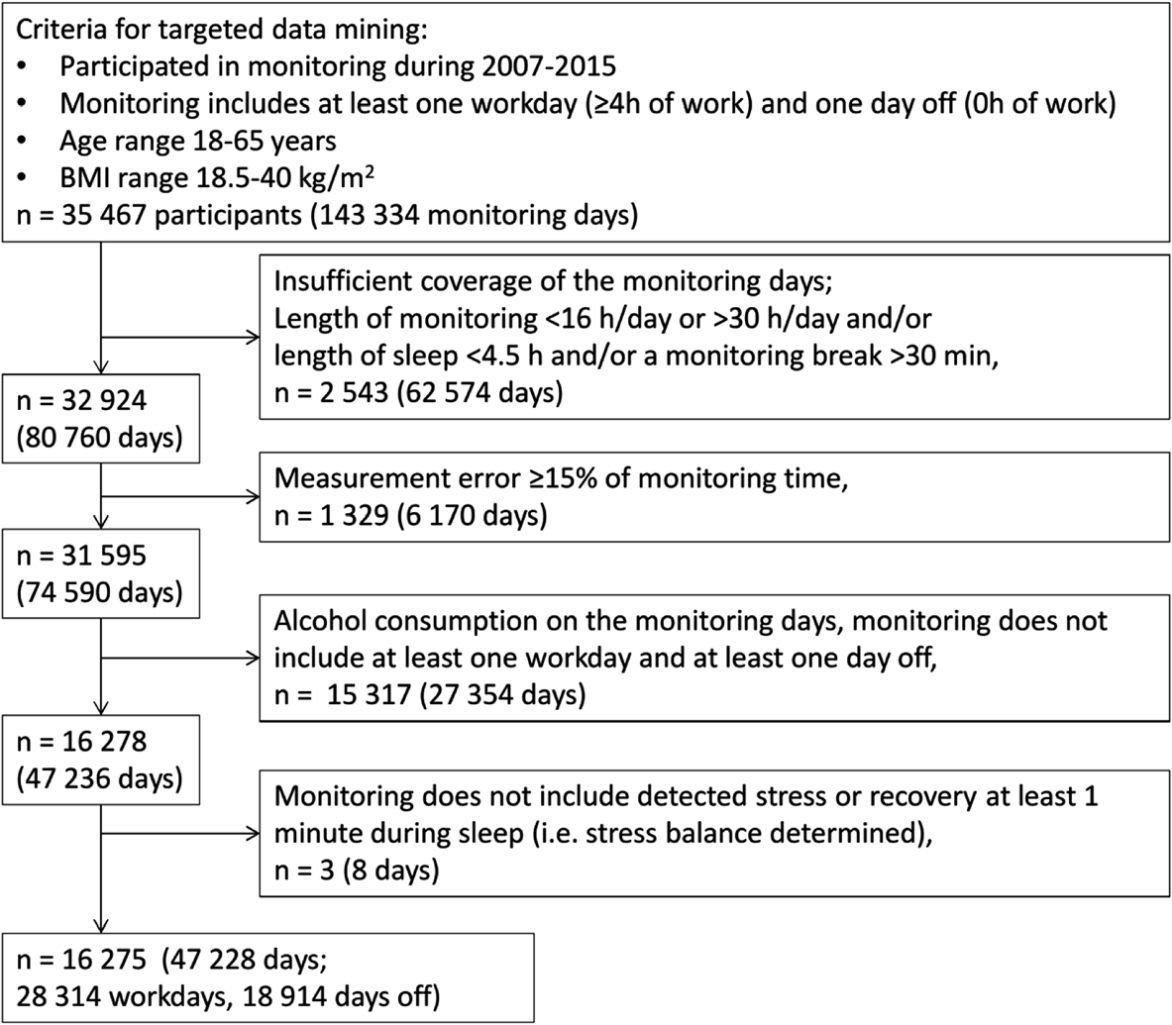
Flow of the participants and measurement days included in the analysis (BMI, body mass index)

The novel methodology used to determine the participants’ stress, recovery and level of weekly PA, was based on HRV data from beat-to-beat R-R interval recordings. These recordings were voluntarily performed on employees as a part of the preventive occupational health care programs provided by their employers between 2007 and 2015. The clinical purpose of these measurements is presented comprehensively in a previous paper by Mutikainen et al. [31]. The data recordings used in the previous study were gathered between 2007 and 2013, with a study population size of 9554. These data were used in the present study, supplemented with recordings from 2014 to 2015. The study had a further inclusion criterion of a minimum of 4.5 h beat-to-beat R-R interval recording during sleep after a workday. Another inclusion criterion was the availability of R-R interval data, including at least one workday (≥4 h of work) and one day off, with a measurement period of 16–30 h/day (from wake-up to wake-up). Participants who had consumed alcohol on the monitoring days were excluded. Information about workdays, working hours, days off and sleep periods was obtained from diaries that the participants were requested to keep during the measurement period. The analyzed data consisted of successfully recorded (measurement error <15 % and <30-min recording break) days. The flow of the participants included in the analysis is presented in Fig. 1.

### HRV-based assessment of PA, stress and recovery

Ambulatory beat-to-beat R-R interval data were used to determine the amount and intensity of PA, stress and recovery. Using the Firstbeat Bodyguard device (Firstbeat Technologies Ltd., Jyväskylä, Finland), real-life R-R interval data were recorded, usually over 3 days (typically two workdays and one day off) and analyzed using Firstbeat Analysis Server software (version 6.3, Firstbeat Technologies Ltd.), which included a powerful artifact detection and correction feature for irregular ectopic beats and signal noise. The software calculates HRV indices second-by-second using the short-time Fourier transform method, and calculates HR- and HRV-derived variables of respiration rate, oxygen consumption, on-off kinetics (increasing or decreasing HR), and parameters describing excess post-exercise oxygen consumption using neural networks. Thereafter, the software divides the measurement data into coherent data segments and categorizes these segments into different physiological states, such as PA of different intensities, stress and recovery [32–34], by taking into account individual characteristics (e.g. individual levels and scales of HR and HRV, and the individual relationships between HRV and autonomic control) [35]. The categorization of the data is described in Additional file 1: Table S1. More information about this analysis method is available in a paper by Firstbeat Technologies Ltd. [36].

#### Detection of stress and recovery variables

After data categorization, the HRV-based variables describing the amount and intensity of stress and recovery on workdays were detected. Stress percentages (i.e. proportions of stress reactions, during the day and during working hours) and stress balance (ratios between recovery and stress reactions during sleep) describe the amount of stress and recovery, respectively. The variables describing the intensity (i.e. magnitude of recognized reactions) of stress and recovery were stress index (during the day) and recovery index (during sleep), respectively. These variables and their calculations are presented in Additional file 1: Table S1. The correlation coefficient between two consecutive workdays varied from 0.74 to 0.88 for the traditional HRV variables, from 0.64 to 0.93 for HRV-derived variables of stress, and from 0.42 to 0.49 for HRV-derived variables of recovery during sleep.

#### Calculation of weekly PA

Background information about age, sex, self-reported height and weight, and self-reported PA class [37] modified from Ross and Jackson [38], was collected in conjunction with R-R interval recordings using questionnaires. Background information was used to estimate maximal HR [39] and maximal VO_2_ [40] which were then used in the estimation of VO_2_. The maximal HR used for further calculations was corrected accordingly if a period with HR higher than the estimated maximal was found from the recording. From the second-by-second VO_2_ estimations, each participant’s mean VO_2_ for each minute of the measurement day was calculated. The minute-by-minute VO_2_ estimations were then converted to multiples of the resting metabolic rate (MET) by dividing the VO_2_ values by 3.5. The total number of 1-min segments within the following thresholds: moderate PA 3 to <6 METs and vigorous PA ≥6 METs, during each measurement day (including days off), were calculated. Continuous bouts of PA lasting for ≥10 min were included in the estimation of weekly PA. These continuous bouts of PA were calculated separately for workdays and days off, and, if the measurement period included two or more workdays or days off, an average was calculated. The activity minutes score for each day (moderate PA minutes + vigorous PA minutes × 2) was calculated. Thereafter, the amount of PA was extrapolated using the following formula: PA minutes per week = (5 × mean workday activity score) + (2 × mean day-off activity score). These calculations have been previously described in more detail [31]. Based on the weekly PA minutes, the participants were divided into the following PA groups: inactive (0 min), low (0 < 150 min), medium (150–300 min) and high (>300 min).

### Assessment of body composition

BMI was calculated from the self-reported weight and height (kg/m^2^). The participants were then divided into the following groups: normal weight (18.5 to <25 kg/m^2^), overweight (25 to <30 kg/m^2^) and obese (30–40 kg/m^2^).

### Analysis

**Fig. 2:**
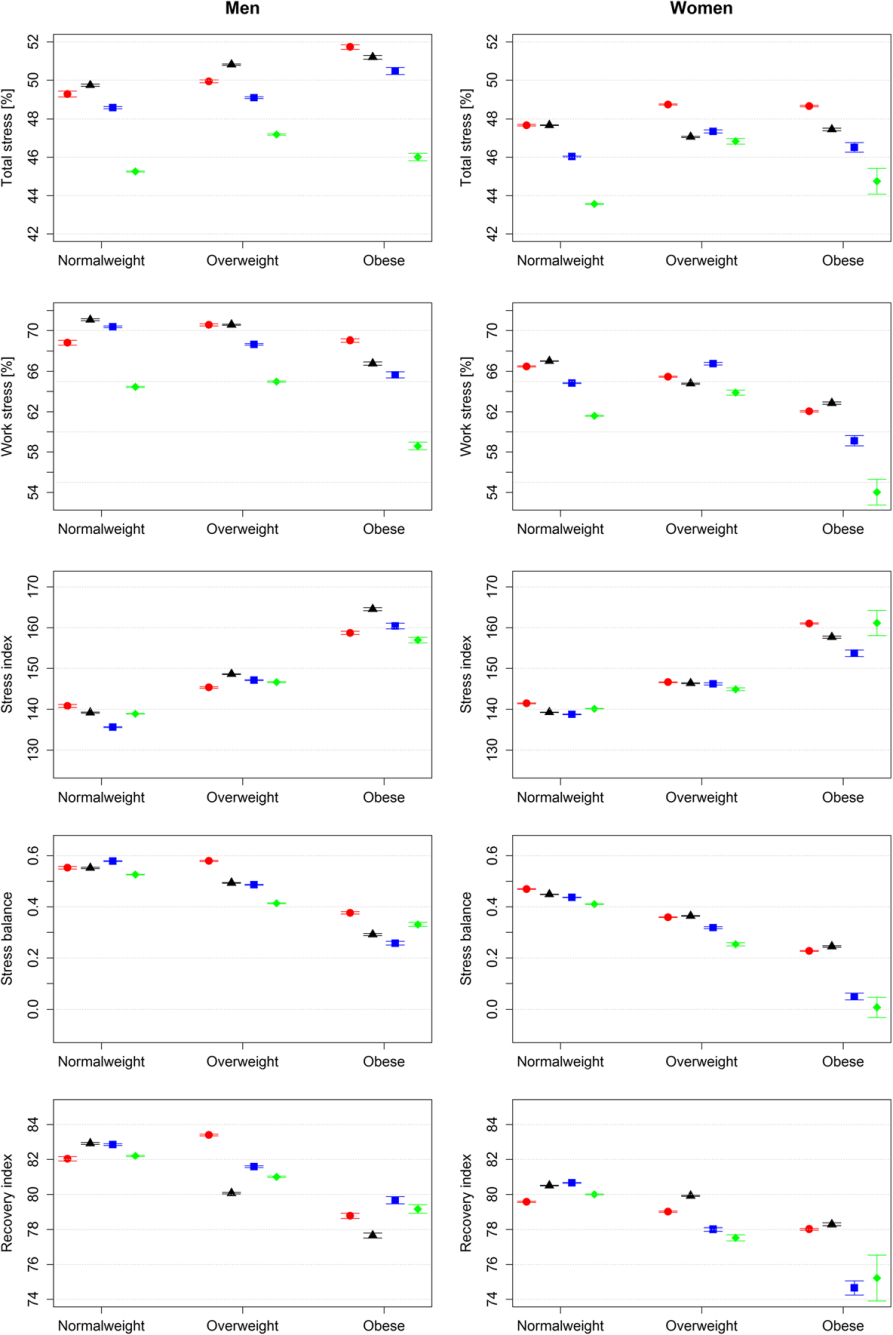
Stress and recovery by physical activity and body mass index groups with age-controlled mean values and 99 % CIs for output variables. Physical activity groups: inactive (0 min/week), *red* ●; low (0 < 150 min/week), *black* ▲; medium (150–300 min/week), *blue* ■; high (>300 min/week), *green* ♦

Data processing and statistical analysis were performed using R 3.2.2 version (R Foundation for Statistical Computing). P-values were two-sided and a p-value of <0.05 was considered statistically significant. Because of the size of the data, 99 % confidence intervals (CIs) were determined (Fig. 2) instead of conventional 95 % CIs.

The main outcome variables of the study were stress percentage and stress index, calculated for the whole day, stress percentage calculated for working hours, and stress balance and recovery index calculated for sleep. These variables were derived from the beat-to-beat R-R interval recordings on workdays. For a more detailed description of the variables see Additional file 1: Table S1. In addition, HR and traditional HRV parameters, including RMSSD and the LF/HF ratio, were calculated from the beat-to-beat R-R interval recordings on workdays. These variables were calculated separately for waking hours and sleep, and RMSSD was calculated using a 5-min window. If the measurement period of a subject included two or more workdays, an average was calculated and the mean values of the outcome variables were used in the analysis.

For the descriptive statistics, the means and standard deviations of the outcome variables were calculated separately for men and women, and stratified based on PA, BMI and age. Differences in the outcome variables between PA, BMI and age groups were tested using the Kruskal-Wallis test. The results are shown in Additional file 2: Tables S2-S4. To show the effects of BMI and PA group on the HRV-based stress and recovery variables, the age-controlled mean values and 99 % CIs for the HRV-based stress and recovery variables, by BMI and PA group, are presented in Fig. 2.

Linear models were employed to study the effects of PA group, BMI and age on HRV-based stress and recovery variables. In the models, age and BMI were incorporated as continuous predictor variables and objectively measured PA group was incorporated as a categorical predictor variable. The models were generated separately for men and women. The reference value for age was set to 18 years and for BMI to 18.5 kg/m^2^. A simple linear least squares regression model (procedure *lm* in R) was applied to predict the stress percentage during the day. As confirmed by visual inspection, the assumption of linear regression considering the normal distribution of the residuals was not fulfilled for stress percentage during working hours, stress index and recovery index. Thus, a Box-Cox transformation was applied on these dependent variables [41]. The Box-Cox coefficient was determined by maximizing the log-likelihood function and was rounded to two decimal places before transformation. Tobit regression model (procedure *vglm* using iteratively reweighted least squares in R) was applied for modeling stress balance with a fixed lower and upper limit of −1 and 1, respectively. The interactions of the predictors were not included in the final regression models because the coefficient of determination for the interaction models was only a few percentage points greater than for the simple models.

## Results

The total number of workdays included in the analysis was 28 314, with measurements obtained from 16 275 participants (men 6863; women 9412). The participants’ characteristics are shown in Table 1. The participants’ mean age was 44.8 years (44.5 years for men; 45.0 years for women) and the mean BMI was 26.0 kg/m^2^ (26.6 kg/m^2^ for men; 25.5 kg/m^2^ for women). The participants’ mean self-reported activity class was 4.8 (4.9 for men; 4.8 for women) indicating that, on average, the participants were involved in PA 2–3 times per week and their total weekly PA was about 2 h. The mean weekly minutes of objective monitoring-based PA was 186 (246 for men; 142 for women). The mean weekly minutes of PA in the group of low PA was 78 for men and 74 for women, in the group of medium PA 222 for men and 215 for women, and in the group of high PA 545 for men and 496 for women. The number of participants in the PA, BMI, and age groups is presented in Additional file 3: Table S5.

Differences in outcome variables between PA groups (Additional file 2: Table S2) were statistically significant except for LF/HF ratio during waking hours and sleep, stress balance in men, and HR and stress balance in women. For both men and women, the high PA group had the highest RMSSD (during waking hours and during sleep) and recovery index, and the lowest stress percentage (during the day and during working hours) and stress index.

Differences in outcome variables between BMI groups were statistically significant for both men and women (Additional file 2: Table S2). Normal-weight individuals had the highest RMSSD (both during waking hours and during sleep), stress balance and recovery index, the lowest stress percentage during the day and the lowest stress index. Stress percentage during working hours was lowest in obese individuals.

In both sexes, differences in outcome variables were statistically significant between age groups, except for HR during sleep in women (Additional file 2: Table S3). The youngest age group (18–30 years) had the highest RMSSD (both during waking hours and during sleep), stress balance and recovery index, and the lowest stress index. Stress percentages during the day were lowest in the youngest age group in women, and in the oldest age group (51–65 years) in men. Stress percentages during working hours were lowest in the oldest age group in both sexes.

Figure 2 shows the effect of PA and BMI group on the stress and recovery variables with the effect of age controlled. The high PA group had the lowest mean stress percentage during the day and during working hours in all three BMI groups, after adjustment for age (Fig. 2). Mean stress index values increased as the BMI group changed from normal weight to overweight and overweight to obese, regardless of the PA group. In addition, regardless of the PA group, obese individuals had the lowest stress balance and recovery index.

The linear model results are shown in Table 2. Medium (*P* < 0.05) and high (*P* < 0.001) PA groups, lower BMI (*P* < 0.001), and older age (*P* < 0.001) were associated with lower stress percentages during the day. Medium (*P* < 0.05) and high (*P* < 0.001) PA level, higher BMI (*P* < 0.001), and older age (*P* < 0.001) were associated with lower stress percentages during working hours. Stress percentage results during the day and during working hours were similar for men and women. Higher BMI (*P* < 0.001) and older age (*P* < 0.001) were associated with higher stress index, both in men and in women. In addition, medium (*P* < 0.01) and high (*P* < 0.01) PA were associated with lower stress index in women.

**Table 2:** Results of the linear models

	Men					Women				
	Parameter Estimate	95 % Cl Lower	Upper	*P* value	Variance explained (%)	Parameter Estimate	95 % Cl Lower	Upper	*P* value	Variance explained (%)
Stress (%), 24 h ^a^					2.356^h^					1.476 ^h^
Intercept	52.3153	50.7730	53.8575	<0.001		50.0658	48.9790	51.1527	<0.001	
Age (18 years = 0)	−0.1263	−0.1600	−0.0926	<0.001	0.780^i^	−0.0869	−0.1151	−0.0587	<0.001	0.387 ^i^
BMI (18.5 kg/m^2^ = 0)	0.1541	0.0580	0.2502	0.002	0.144 ^i^	0.0909	0.0264	0.1553	0.006	0.081 ^i^
Physical activity level (inactive = 0)					1.586 ^i^					1.050 ^i^
Low physical activity class	0.3235	−0.6903	1.3372	0.53		−0.8892	−1.5471	−0.2313	0.008	
Medium physical activity class	−1.1405	−2.2144	−0.0667	0.04		−1.9539	−2.7608	−1.1470	<0.001	
High physical activity class	−3.9695	−5.0062	−2.9328	<0.001		−4.3772	−5.2620	−3.4923	<0.001	
Stress (%), working hours ^b^					4.152 ^h^					1.721 ^h^
Intercept	807.3480	777.0476	837.6483	<0.001		677.8091	656.0738	699.5443	<0.001	
Age (18 years = 0)	−4.8729	−5.5355	−4.2103	<0.001	2.942 ^i^	−2.66482	−3.22838	−2.10127	<0.001	0.905 ^i^
BMI (18.5 kg/m^2^ = 0)	−4.6640	−6.5515	−2.7766	<0.001	0.341 ^i^	−4.54067	−5.82984	−3.25149	<0.001	0.504 ^i^
Physical activity level (inactive = 0)					1.590 ^i^					0.584 ^i^
Low physical activity class	−5.5094	−25.4261	14.4073	0.59		−3.40975	−16.5664	9.746938	0.61	
Medium physical activity class	−24.7847	−45.8819	−3.6874	0.021		−17.9339	−34.0695	−1.79829	0.030	
High physical activity class	−85.2896	−105.6576	−64.9215	<0.001		−61.7878	−79.4833	−44.0923	<0.001	
Stress index, 24 h ^c^					27.113 ^h^					27.448 ^h^
Intercept	1.2406	1.2403	1.2409	<0.001		1.2414	1.2412	1.2417	<0.001	
Age (18 years = 0)	0.0001	0.0001	0.0001	<0.001	21.992 ^i^	0.0001	0.0001	0.0001	<0.001	19.655 ^i^
BMI (18.5 kg/m^2^ = 0)	0.0002	0.0001	0.0002	<0.001	3.819 ^i^	0.0001	0.0001	0.0001	<0.001	3.031 ^i^
Physical activity level (inactive = 0)					0.115 ^i^					0.145 ^i^
Low physical activity class	0.0002	0.0000	0.0004	0.024		−0.0001	−0.0003	0.0000	0.031	
Medium physical activity class	0.0000	−0.0002	0.0002	0.91		−0.0002	−0.0004	−0.0001	0.002	
High physical activity class	0.0001	−0.0001	0.0003	0.47		−0.0003	−0.0004	−0.0001	0.002	
Stress balance, sleep ^e^					3.669 ^h^					3.244 ^h^
Intercept1	0.9975	0.9287	1.0663	<0.001		0.6166	0.5672	0.6661	<0.001	
Intercept2	−0.5292	−0.5503	−0.5082	<0.001		−0.5537	−0.5705	−0.5368	<0.001	
Age (18 years = 0)	−0.0066	−0.0081	−0.0052	<0.001	1.153 ^i^	−0.0007	−0.0019	0.0006	0.31	0.006 ^i^
BMI (18.5 kg/m^2^ = 0)	−0.0273	−0.0315	−0.0231	<0.001	2.179 ^i^	−0.0253	−0.0282	−0.0224	<0.001	3.080 ^i^
Physical activity level (inactive = 0)					0.445 ^i^					0.233 ^i^
Low physical activity class	−0.0715	−0.1162	−0.0269	0.002		−0.0126	−0.0423	0.0172	0.41	
Medium physical activity class	−0.0799	−0.1272	−0.0326	<0.001		−0.0572	−0.0937	−0.0207	0.002	
High physical activity class	−0.1327	−0.1784	−0.0870	<0.001		−0.0822	−0.1222	−0.0421	<0.001	
Recovery index, sleep ^f^					9.685 ^h^					3.297 ^h^
Intercept	592375.4298	573358.1497	611392.7100	<0.001		457913.8301	444648.7930	471178.8673	<0.001	
Age (18 years = 0)	−5092.7330	−5508.5947	−4676.8712	<0.001	7.753 ^i^	−2100.7061	−2444.6430	−1756.7692	<0.001	1.501 ^i^
BMI (18.5 kg/m^2^ = 0)	−4825.0722	−6009.6783	−3640.4662	<0.001	0.921 ^i^	−4038.5841	−4825.3668	−3251.8014	<0.001	1.065 ^i^
Physical activity level (inactive = 0)					0.154 ^i^					0.026 ^i^
Low physical activity class	−19214.5686	−31714.7934	−6714.3437	0.003		1051.0555	−6978.4656	9080.5766	0.80	
Medium physical activity class	−6153.1124	−19394.2693	7088.0445	0.36		−4452.2347	−14299.7786	5395.3093	0.38	
High physical activity class	−9653.5430	−22437.0465	3129.9606	0.14		−5732.9247	−16532.4796	5066.6302	0.30	

*BMI* body mass index

^a^ Linear regression

^b^ Box-Cox linear regression using transformation coefficient of 1.62

^c^ Box-Cox linear regression using transformation coefficient of −0.79

^e^ Tobit regression

^f^ Box-Cox linear regression using transformation coefficient of 3.18

^h^ The proportion of variance explained by the whole model

^i^ The proportion of variance explained by the predictor variable. Calculated as the difference between the proportion of variance explained by the whole model and the proportion of variance explained by a model including all the predictor variables, except for the predictor in question

Medium (*P* < 0.01) and high (*P* < 0.001) PA, and higher BMI (*P* < 0.001) were associated with lower stress balance, both in men and in women. Moreover, older age was associated with lower stress balance in men (*P* < 0.001). Higher BMI and older age were associated with lower recovery index, in men and in women (*P* < 0.001). BMI explained the highest proportion of variance in stress balance (2.2 % for men and 3.1 % for women) compared with PA and age.

## Discussion

The purpose of this study was to investigate the amount and intensity of objective HRV-based stress and recovery on workdays. The sample group comprised 16 275 Finnish employees, who had participated in beat-to-beat R-R interval recording as a part of lifestyle counseling in the course of their everyday lives between 2007 and 2015. More specifically, the relationships between PA, BMI, and HRV-based stress and recovery were investigated. For both sexes, a high level of PA and lower BMI were associated with lower amounts of stress on workdays. Additionally, the results showed that both high PA and higher BMI were associated with a lower amount of recovery during sleep. Additional PA (above the generally recommended aerobic PA level of over 150 min of moderate PA per week), was associated with the additional health benefits of a low amount of HRV-based stress on workdays and during working hours. Lower BMI was associated with better recovery during sleep, expressed by a greater amount and magnitude of recovery reactions (i.e. quality of recovery). This suggests that PA in the long term resulting in improved physical fitness has a positive effect on recovery, even though high PA may disturb recovery during the following night. The results of the present study showing an association of BMI and objectively measured PA with HRV-based stress during the workday are in line with previous studies.

The finding of the present study on the association of high PA with low HRV-based stress on workdays is in line with previous studies. Both moderate and vigorous PA are found to be associated with higher HRV [29]. Additionally, PA has been found to have positive effects on subjective stress. For instance, Birdee et al. [42] found that, among a large group of employees, physically active employees reported less difficulty coping with stress, more happiness and a higher rate of competency than inactive employees. Our previous study used the same measurement method to assess stress as in the present study, and found higher PA and physical fitness were associated with lower stress among men [43]. However, to our knowledge, this study is unique in its focus on the additional health benefits from PA exceeding the recommended level, in the context of stress. The results showed that PA level affects stress percentage more than BMI, especially in women, and the decrease in the amount of stress following a change from inactivity to high PA appears to be impossible to achieve by weight loss alone. When stress percentage was calculated without the time spent on PA, the association between higher PA with lower stress percentage remained (data not shown).

The present findings of an association between lower BMI and lower amount of stress on workdays, and an association of higher BMI with lower amount of stress during working hours, are also in line with previous studies. Furthermore, an additional analysis (data not shown) showed that having a higher BMI was associated with a higher amount of PA during working hours. Previously, HRV profiles were found to be relatively poor among obese individuals [44] and improved after weight loss [45]. Previous studies also suggest that individuals with lower socioeconomic status are more likely to be obese and more likely to be in physically active employment than their counterparts with higher socioeconomic status [46, 47]. So, time spent in PA leads to less time for other physiological body states, such as stress, during working hours. Another possible explanation for these findings is that among obese individuals, the physiological state of the body is detected as PA instead of stress, as HR increases and HRV decreases easily. Therefore, caution is required when interpreting the results. Previously, the association of BMI with stress has been studied using mainly subjective methods. For instance, Nyberg et al. [16] found both obesity and being underweight to be associated with high levels of work-related stress [16], independent of sex. Additionally, employees of normal weight report the lowest prevalence of emotional exhaustion and chronic psychological complaints compared with underweight, overweight and obese individuals [17]. In general, the evidence is weak and inconsistent for associations of psychosocial factors at work with weight-related outcomes [22]. However, based on previous [43–45] and present results, the association of obesity with HRV and HRV-based stress seems to be consistent.

The group of high active, consisting largely of young and normal-weight individuals, had the best quality of recovery during sleep when age and weight were not taken into account. The linear models revealed that BMI and age explained greater proportion of variance in the quality of recovery than the level of PA. Further, the linear models showed that the non-significant association of high PA with lower quality of recovery during sleep was negative. Additionally, high PA was significantly associated with lower amount of recovery during sleep. This finding may be explained by the estimation of PA level occurring on the same days that stress and recovery during sleep were determined. We did not take into account the timing of PA in the analysis of the present study. The findings of Myllymäki et al. [48] suggest that vigorous late-night exercise may have effects on cardiac autonomic control of heart during the first sleeping hours. They found higher nocturnal HR after the exercise day compared to the control day but no differences between the days in nocturnal HRV. Additionally, previous literature suggests that PA during working hours and leisure-time may show different effects on cardiac autonomic regulation. High PA during working hours has been found to be associated with poor cardiovascular health, including reduced HRV [49]. Recovery of HRV is also dependent on training background, and type, intensity and duration of exercise [50]. The lower the physical fitness and the higher the intensity of exercise, the slower the recovery of HRV after exercise [51]. Hynynen et al. [52] reported that even an exercise that was perceived as light and easy may have prolonged effects on nocturnal HRV during the following night. Our previous study with a smaller study population used a subjective method to assess PA; using laboratory conditions to assess physical fitness, our previous results showed that physical fitness was associated with better recovery during sleep, even though PA was not [43]. In line with this Pietilä et al. [53] found good physical fitness to be associated with good recovery, even though PA was found to disturb the recovery of the following night. It appears that PA on the same day may disturb nighttime recovery, but in the long term, PA and good fitness enhance recovery during sleep. This is supported by our additional analysis (data not shown), which showed higher recovery in a day without PA compared with a day with PA, among high-PA individuals. Further, the present finding of an association of lower BMI with a higher amount and better quality of recovery supports the idea that good fitness enhances recovery during sleep. However, the effect of the timing of PA on HRV-based recovery during following night should be studied further.

The present findings suggest that, although older individuals are not stressed as often, their stress reactions are stronger and recovery is weaker than their younger counterparts. Weaker recovery among older individuals was expected, as it is known that aging reduces HRV [24]. Compared with PA and BMI, age was most strongly associated with the amount of stress during working hours, and intensity of stress and recovery reactions. These results suggest that recovery of older individuals is weakened. However, the findings of Soares-Miranda et al. [30] showing both cross-sectional and longitudinal association of PA with more favorable HRV among older adults emphasizes the importance of PA among older individuals. These findings should be considered for instance in policymaking when planning to lengthen working careers.

The results were similar between men and women. The variances explained by the linear models were mostly slightly higher for men. The men in this study had a slightly higher amount of stress than women during the whole workday and during working hours. Men also had a higher intensity of stress reactions and a lower amount and quality of recovery during sleep compared with women. This finding is in line with previous evidence that men have stronger physiological responses to psychological stress than women, including greater cardiovascular activation [18].

This study has strengths and weaknesses. While the measurement method may have had a significant impact on the measured PA levels, a strength of this study is that the weekly PA amount was calculated based on the objective measurement of PA periods lasting over 10 min. The validated ambulatory beat-to-beat R-R interval-based method [31, 54] used to assess the amount and intensity of PA has been shown to provide more accurate estimates of the intensity of PA than HR information [54, 55]. Even though the participants were informed to continue with normal daily living under the wellness assessment, individuals may have a tendency to be more active than usual during this type of short-time assessment. At least 5 consecutive days of pedometer monitoring has been suggested to achieve reliable and valid 1-year PA estimates [56]. However, another study suggests that three days would be sufficient to achieve valid results [57]. The existing literature indicates a need for valid, accurate and reliable measures of PA for assessing current and changing PA levels and the relationships between PA and health outcomes [58].

We used a novel HRV-based method to assess the amount and intensity of stress and recovery. HRV has been suggested as a feasible stress assessment method [59–61], and the stability of 24-h recording is high [24]. In our study, the sustainability of the HRV and HRV-based measures of stress and recovery between 2 consecutive days was quantified, and all the correlations were found to be statistically significant. The method used in the present study has been validated against neuroendocrine responses to stress, and the indicators of stress and recovery during sleep have been found to be associated with free salivary cortisol response after awakening [62]. Additionally, the method has been utilized in previous studies [43, 63–65] and the findings of these studies further support the validity and reliability of this HRV-based method. For instance, previous studies have found an association of higher HRV-based stress and lower recovery with higher perceived stress [63, 64]. Although traditional HRV measures are required to assess quality and clinical correlates of the recordings, these new ways of presenting findings improve the usability of HRV recordings in health promotion. Traditional HRV measures are not included in the main study analyses. However, the descriptive statistics show the similarity between the traditional HRV measures and the HRV-based stress and recovery variables. HRV-based methods that take individual characteristics and dynamic changes in cardiac autonomic activity into account and provide easily understandable variables of stress and recovery can be informative and suitable measures for field and clinical conditions. Individual written feedback together with verbal feedback and discussion of the HRV recording results would be optimal (an example of the feedback the participants received is shown in Additional file 4: Figure S1). It should be noted here that the method we used did not distinguish eustress from distress. However, division into these two types of stress may be impractical because of similar physiological responses to both stress forms.

The major strength of the present study is the very large sample, which included both non-manual and manual labor employees. The study sample was not a random sample from the Finnish population, but a real-life sample of Finnish employees who voluntary performed beat-to-beat R-R interval recording as a part of the preventive occupational health care programs provided by their employers. Even though the participants of the present study may represent a group of employees who are more interested about their health than the average person, their BMI profiles were similar to ordinary working-aged Finnish people [66], except for the individuals with BMI over 40 were excluded from the present study. Thus, the findings of this study are generalizable to ordinary Finnish employees. Nonetheless, it is a weakness of the present study that as it was a real-life/data-mining type study, we did not have detailed individual information about the participants, including the information about the profession or socioeconomic status of the participants. Additionally, the fact that the information about weight and height (needed for BMI calculation) was based on self-reports may have yielded an underestimation of BMI in the study sample [67]. Most of the participants were apparently healthy; however, the inclusion of individuals with chronic diseases and/or on medications may have had an effect on HRV. However, our large sample size should have compensated for these inclusions, leading to statistically significant results. The use of real-life data was a strength of the study; however, daytime stress is affected by many confounding factors [24], very few of which were controlled for in our analysis. Participants who had consumed alcohol on the monitoring days were excluded from the analyses of the present study. Unfortunately, we did not have information for example about participants’ smoking or caffeine consumption. Clearly, outside factors appear to have affected HRV-based stress because the explanation ratios of the linear models were rather small. In summary, the large real-life study sample of the present study can be considered as either a strength or a weakness depending on the perspective. For instance, in future it would be interesting to study the association of socioeconomic status with HRV-based stress and recovery by taking into account the effect of the level of PA.

This study used novel, validated [43, 62–64] HRV-based technology to assess stress, recovery and PA in real-life. The results suggest that high PA and lower BMI are associated with a lower amount of stress on workdays independently of age and sex. Additionally, the results suggest that having a lower BMI is associated with lower intensity of stress reactions on workdays, and a higher amount and better quality of recovery during sleep. This, together with existing evidence, suggests that long-term PA, resulting in improved physical fitness, has a positive effect on recovery, even though high PA was associated with a lower amount of recovery on the following night. In summary, the present results support the beneficial effects of PA on health. However, owing to the cross-sectional study design, it is not possible to draw conclusions about the direction of the associations. Previous literature suggests that the association may be reciprocal; that is, inactivity may cause stress or stress may be a factor that leads to inactivity. Overall, most of the literature finds that the experience of stress impairs efforts to be physically active [68], even though PA is beneficial in stress management [65]. More research on the causal relations between PA and HRV-based stress and recovery is needed. Randomized controlled trials investigating the effect of increasing different types of PA and timing of PA are warranted.

## Conclusions

The results provide important information about the associations of objectively measured PA and body weight with objectively measured physiological stress in Finnish employees. This information could be used in future policymaking and focused upon by employers. Although the beneficial effects of PA on health are well documented, these results may be beneficial by, for example, increasing employer willingness to invest greater resources in increasing the PA of employees.

## Abbreviations

ANS, autonomic nervous system; BMI, body mass index; HF, high frequency; HR, heart rate; HRV, heart rate variability; LF, low frequency; MET, multiple of the resting metabolic rate; PA, physical activity; RMSSD, root mean square of successive R-R intervals; VO_2_, oxygen uptake; VO_2max_, maximal oxygen uptake

## Additional files

Additional file 1: Table S1. Detection of heart rate variability-based stress and recovery. (DOCX 21 kb) Additional file 2: Table S2. Characteristics (mean ± SD) of the measures derived from beat-to-beat R-R interval recording, by physical activity groups. Table S3. Characteristics (mean ± SD) of the measures derived from beat-to-beat R-R interval recording, by body mass index groups. Table S4. Characteristics (mean ± SD) of the measures derived from beat-to-beat R-R interval recording, by age groups. (DOCX 31 kb) Additional file 3: Table S5. The number of participants by age, physical activity and body mass index groups. (DOCX 21 kb) Additional file 4: Figure S1. An example (1 of the 3 days) of the feedback from the wellbeing assessment of the participants. (PDF 262 kb)
