# Supplementary material for: Physical activity, body mass index and heart rate variability-based stress and recovery in 16 275 Finnish employees: a cross-sectional study
Source: BMC Public Health. 2016 Aug 2;16:701. doi: 10.1186/s12889-016-3391-4 (PMC4971625; doi:10.1186/s12889-016-3391-4)
Supplement: Additional file 1: Table S1. — Detection of heart rate variability-based stress and recovery. (DOCX 21 kb) [file 12889_2016_3391_MOESM1_ESM.docx]

| Table S1 Detection of heart rate variability−based stress and recovery | |
| --- | --- |
| To detect stress and recovery, second−by−second indices reflecting activities of the sympathetic and parasympathetic nervous systems were calculated. The traditional time domain and frequency domain HRV variables needed for state detection include RMSSD, HF (0.15–0.40 Hz) and LF (0.04–0.15 Hz) components of HRV, and HRV−based respiration rate. The software categorizes the data by taking into account individual characteristics (e.g. the individual levels and scales of HR and HRV, and the individual relationships between HRV and autonomic control).   - **Stress** is detected when sympathetic activity of the ANS is dominating without metabolic requirements caused by physical activity. Variables related to stress detection include HF and LF components of HRV, respiration rate, and HR. During the stress state, HR is elevated, HRV is reduced, and the frequency distribution of HRV is inconsistent because of changes in respiratory period. - **Recovery** is detected when parasympathetic activity of ANS is dominating. During recovery, HR is low and HRV is high and regular. | |
| ***HRV−based stress variables*** |  |
| Stress percentage | The percentage of 1−minute segments classified as stress during the 24−hour period. |
| Stress percentage, working hours | The percentage of 1−minute segments classified as stress during working hours. |
| Stress index | The mean value of the magnitude of stress reactions in 1−minute segments classified as stress, during the 24−hour period. |
| ***HRV−based recovery (sleep) variables*** |  |
| Stress balance | The difference between the total time of 1−minute segments classified as recovery during sleep and the total time of 1−minute segments classified as stress, divided by the sum of the total time of 1−minute segments classified as recovery during sleep and the total time of 1−minute segments classified as stress.  Values from 0.5 to 1 indicate good recovery; values from 0 to 0.5 indicate moderate recovery; and values from 0 to −1 indicate weak recovery. |
| Recovery index | The mean value of the magnitude of recovery reactions in 1−minute segments classified as recovery, during the 4−hour window starting 30 minutes after going to bed. |
| ANS, autonomic nervous system  HF, high frequency  HRV, heart rate variability  HR, heart rate  LF, low frequency  RMSSD, root mean square of successive R−R intervals | |
