# Supplementary material for: Physical activity, body mass index and heart rate variability-based stress and recovery in 16 275 Finnish employees: a cross-sectional study
Source: BMC Public Health. 2016 Aug 2;16:701. doi: 10.1186/s12889-016-3391-4 (PMC4971625; doi:10.1186/s12889-016-3391-4)
Supplement: Additional file 2: Table S2. — Characteristics (mean ± SD) of the measures derived from beat-to-beat R-R interval recording, by physical activity groups. Table S3. Characteristics (mean ± SD) of the measures derived from beat-to-beat R-R interval recording, by body mass index groups. Table S4. Characteristics (mean ± SD) of the measures derived from beat-to-beat R-R interval recording, by age groups. (DOCX 31 kb) [file 12889_2016_3391_MOESM2_ESM.docx]

| Table S2 Characteristics (mean ± SD) of the measures derived from beat−to−beat R−R interval recording, by physical activity groups | | | | | | | | | | |
| --- | --- | --- | --- | --- | --- | --- | --- | --- | --- | --- |
| Variable | Men (n=6863) | | | |  | Women (n=9412) | | | |  |
|  | Inactive  (n=1117) | Low  (n=1986) | Medium  (n=1591) | High  (n=2169) | *P* | Inactive  (n=3055) | Low  (n=3175) | Medium  (n=1709) | High  (n=1473) | *P* |
| HR,  waking hours | 74.3±8.6 | 76.4±8.5 | 76.7±8.6 | 79.1±9.7 | <0.001 | 78.8±8.2 | 80.4±8.1 | 81.2±8.3 | 83.6±9.2 | <0.001 |
| HR,  sleep | 59.7±7.5 | 60.1±8.1 | 58.9±7.9 | 59.0±8.6 | <0.001 | 63.4±7.7 | 63.2±7.5 | 62.9±7.6 | 62.8±8.3 | 0.088 |
| RMSSD,  waking hours | 23.7±10.6 | 24.3±10.4 | 26.4±11.4 | 26.7±11.4 | <0.001 | 22.5±9.6 | 24.0±9.8 | 25.6±10.5 | 27.2±11.0 | <0.001 |
| RMSSD,  sleep | 35.8±18.1 | 37.2±17.7 | 41.3±20.1 | 43.5±21.2 | <0.001 | 34.0±16.1 | 36.2±17.2 | 38.0±18.1 | 42.4±20.2 | <0.001 |
| LF/HF ratio,  waking hours | 3.82±1.79 | 3.90±1.64 | 3.84±1.77 | 3.82±1.63 | 0.053 | 2.93±1.24 | 2.95±1.16 | 3.00±1.30 | 2.93±2.13 | 0.013 |
| LF/HF ratio,  sleep | 2.58±1.53 | 2.57±1.46 | 2.51±1.40 | 2.55±1.49 | 0.531 | 1.73±1.01 | 1.74±0.95 | 1.84±1.04 | 1.76±0.98 | <0.001 |
| Stress (%),  24 hours | 50.0±15.0 | 50.5±13.7 | 49.1±13.7 | 46.4±13.3 | <0.001 | 48.2±13.8 | 47.4±12.3 | 46.5±12.7 | 44.3±12.5 | <0.001 |
| Stress (%),  working hours | 68.5±23.3 | 69.6±21.5 | 69.3±21.3 | 65.1±23.5 | <0.001 | 64.1±22.5 | 65.6±20.6 | 65.3±21.4 | 63.0±22.1 | 0.004 |
| Stress index,  24 hours | 155.1±46.3 | 151.1±44.9 | 142.7±41.3 | 139.1±39.0 | <0.001 | 155.7±46.4 | 145.2±43.4 | 137.9±38.6 | 130.1±37.5 | <0.001 |
| Stress balance,  sleep | 0.49±0.54 | 0.47±0.55 | 0.50±0.53 | 0.48±0.53 | 0.227 | 0.35±0.57 | 0.39±0.52 | 0.38±0.55 | 0.39±0.55 | 0.175 |
| Recovery index,  sleep | 80.6±14.1 | 80.1±15.8 | 82.1±14.2 | 82.2±15.8 | <0.001 | 78.4±15.7 | 79.9±12.6 | 79.9±14.4 | 80.3±14.9 | <0.001 |
| Inactive (0 min/week), Low (0<150 min/week), Medium (150–300 min/week), High (>300 min/week)  Kruskal–Wallis test was used to analyze the between−group differences.  HR, heart rate  RMSSD, root mean square of successive R−R intervals  LF/HF, low frequency/high frequency | | | | | | | | | | |

| Table S3 Characteristics (mean ± SD) of the measures derived from beat−to−beat R−R interval recording, by body mass index groups | | | | | | |
| --- | --- | --- | --- | --- | --- | --- |
|  | Men (n=6863) | | | Women (n=9412) | | |
|  | Normal weight  (n=2475) | Overweight  (n=3353) | Obese  (n=1037) | Normal weight  (n=5113) | Overweight  (n=2765) | Obese  (n=1534) |
| HR, waking hours | 75.5±8.6 | 77.1±9.0 | 80.4±9.5 | 79.8±8.5 | 80.8±8.4 | 82.3±8.5 |
| HR, sleep | 57.6±7.6 | 59.8±8.2 | 62.6±8.2 | 62.0±7.5 | 63.9±7.5 | 65.7±7.7 |
| RMSSD, waking hours | 28.4±11.2 | 24.6±10.7 | 20.8±10.0 | 26.5±10.5 | 22.7±9.3 | 19.9±8.6 |
| RMSSD, sleep | 43.0±19.9 | 38.7±19.6 | 36.6±18.7 | 38.8±18.8 | 34.5±16.2 | 34.1±15.4 |
| LF/HF ratio, waking hours | 3.68±1.52 | 3.92±1.74 | 4.00±1.86 | 2.87±1.18 | 3.01±1.79 | 3.10±1.30 |
| LF/HF ratio, sleep | 2.59±1.47 | 2.59±1.47 | 2.36±1.45 | 1.81±1.01 | 1.76±0.97 | 1.56±0.94 |
| Stress (%), 24 hours | 47.8±13.6 | 49.1±14.1 | 50.2±13.8 | 46.4±12.9 | 47.7±12.9 | 47.9±13.2 |
| Stress (%), working hours | 68.7±22.3 | 68.1±22.4 | 65.3±23.0 | 65.5±21.3 | 64.9±21.3 | 61.3±22.9 |
| Stress index, 24 hours | 134.8±35.0 | 148.8±41.8 | 163.7±54.8 | 136.2±39.0 | 150.0±41.3 | 164.8±53.3 |
| Stress balance, sleep | 0.56±0.50 | 0.48±0.53 | 0.31±0.59 | 0.44±0.52 | 0.34±0.55 | 0.21±0.59 |
| Recovery index, sleep | 83.16±13.81 | 80.93±15.11 | 78.13±17.86 | 80.50±13.57 | 78.78±14.30 | 77.34±16.60 |
| Normal weight (18.5 to <25 kg/m^2^), Overweight (25 to <30 kg/m^2^), Obese (30–40 kg/m^2^).  Kruskal–Wallis test was used to analyze the between−group differences. The difference was statistically significant (P<0.001) for all measures.  HR, heart rate  RMSSD, root mean square of successive R−R intervals  LF/HF, low frequency/high frequency | | | | | | |

| Table S4 Characteristics (mean ± SD) of the measures derived from beat−to−beat R−R interval recording, by age groups | | | | | | | | |
| --- | --- | --- | --- | --- | --- | --- | --- | --- |
|  | Men (n=6863) | | | | Women (n=9412) | | | |
|  | 18−30 yrs  (n=615) | 31−40 yrs  (n=1898) | 41−50 yrs  (n=2198) | 51−65 yrs  (n=2152) | 18−30 yrs  (n=847) | 31−40 yrs  (n=2256) | 41−50 yrs  (n=3206) | 51−65 yrs  (n=3103) |
| HR, waking hours | 79.2±9.0 | 77.5±8.6 | 77.3±9.1 | 75.6±9.2 | 83.4±8.8 | 81.6±8.5 | 80.4±8.3 | 79.1±8.4 |
| HR, sleep | 57.6±7.9 | 58.3±8.0 | 59.9±8.0 | 60.5±8.2 | 62.9±8.3 | 63.4±7.8 | 63.3±7.6 | 62.9±7.5 |
| RMSSD, waking hours | 33.9±12.3 | 30.0±11.5 | 24.0±9.6 | 20.4±8.5 | 31.9±11.4 | 28.5±10.6 | 23.8±9.0 | 19.7±7.9 |
| RMSSD, sleep | 56.5±20.8 | 48.4±21.2 | 37.2±16.3 | 30.4±14.4 | 50.7±21.4 | 42.8±18.4 | 35.5±16.3 | 29.9±13.4 |
| LF/HF ratio, waking hours | 3.15±1.25 | 3.72±1.53 | 4.14±1.70 | 3.86±1.85 | 2.70±2.51 | 2.86±1.23 | 3.04±1.20 | 2.99±1.28 |
| LF/HF ratio, sleep | 1.74±0.90 | 2.20±1.32 | 2.73±1.44 | 2.92±1.59 | 1.40±0.77 | 1.62±0.89 | 1.85±1.03 | 1.86±1.04 |
| Stress (%), 24 hours | 48.0±12.8 | 49.8±13.0 | 49.6±13.7 | 47.3±14.9 | 45.3±12.9 | 47.5±12.6 | 47.9±12.8 | 46.2±13.5 |
| Stress%, working hours | 69.5±21.3 | 72.1±21.0 | 68.8±21.6 | 62.9±23.9 | 65.0±22.0 | 66.9±20.6 | 65.7±21.0 | 61.7±22.6 |
| Stress index, 24 hours | 116.8±24.5 | 127.0±28.6 | 147.4±38.7 | 168.7±48.7 | 116.3±24.4 | 126.4±30.8 | 143.7±37.3 | 167.4±50.1 |
| Stress balance, sleep | 0.58±0.50 | 0.55±0.50 | 0.47±0.53 | 0.40±0.57 | 0.42±0.54 | 0.40±0.55 | 0.37±0.54 | 0.36±0.55 |
| Recovery index, sleep | 85.4±15.5 | 84.5±15.0 | 81.3±14.0 | 77.3±15.5 | 81.6±13.5 | 80.8±15.4 | 79.8±13.5 | 77.7±14.5 |
| Kruskal−Wallis test was used to analyze the between group differences. The difference was statistically significant (P <0.001) for all measures, except for HR (sleep) and stress balance for women, P=0.062 and P=0.001, respectively.  HR, heart rate  RMSSD, root mean square of successive R−R intervals  LF/HF, low frequency/high frequency | | | | | | | | |
