# Supplementary material for: Physical activity, body mass index and heart rate variability-based stress and recovery in 16 275 Finnish employees: a cross-sectional study
Source: BMC Public Health. 2016 Aug 2;16:701. doi: 10.1186/s12889-016-3391-4 (PMC4971625; doi:10.1186/s12889-016-3391-4)
Supplement: Additional file 3: Table S5. — The number of participants by age, physical activity and body mass index groups. (DOCX 21 kb) [file 12889_2016_3391_MOESM3_ESM.docx]

| Table S5 The number of participants by age, physical activity and body mass index groups | | | | | | | | | |
| --- | --- | --- | --- | --- | --- | --- | --- | --- | --- |
| BMI | Physical activity | Men | | | | Women | | | |
|  |  | 18−30 yrs | 31−40 yrs | 41−50 yrs | 51−65 yrs | 18−30 yrs | 31−40 yrs | 41−50 yrs | 51−65 yrs |
| Normal weight  (18.5 to <25 kg/m^2^) |  |  |  |  |  |  |  |  |  |
|  | Inactive | 14 | 66 | 74 | 94 | 54 | 241 | 332 | 420 |
|  | Low | 57 | 188 | 175 | 173 | 142 | 484 | 574 | 498 |
|  | Medium | 78 | 201 | 166 | 174 | 159 | 343 | 399 | 264 |
|  | High | 190 | 315 | 280 | 230 | 292 | 397 | 346 | 168 |
| Overweight  (25 to <30 kg/m^2^) |  |  |  |  |  |  |  |  |  |
|  | Inactive | 15 | 103 | 175 | 257 | 20 | 156 | 374 | 566 |
|  | Low | 43 | 242 | 344 | 414 | 38 | 209 | 389 | 366 |
|  | Medium | 64 | 228 | 257 | 232 | 37 | 97 | 153 | 132 |
|  | High | 100 | 313 | 334 | 232 | 50 | 78 | 62 | 38 |
| Obese  (30–40 kg/m^2^) |  |  |  |  |  |  |  |  |  |
|  | Inactive | 7 | 58 | 116 | 138 | 15 | 117 | 342 | 418 |
|  | Low | 17 | 77 | 129 | 127 | 22 | 93 | 165 | 195 |
|  | Medium | 10 | 56 | 82 | 43 | 6 | 36 | 52 | 31 |
|  | High | 20 | 51 | 66 | 38 | 12 | 5 | 18 | 7 |
|  |  |  |  |  |  |  |  |  |  |
| All |  | 615 | 1898 | 2198 | 2152 | 847 | 2256 | 3206 | 3103 |
| BMI, body mass index  Inactive (0 min/week), Low (0<150 min/week), Medium (150–300 min/week), High (>300 min/week) | | | | | | | | | |
