# Supplementary material for: Physical activity, body mass index and heart rate variability-based stress and recovery in 16 275 Finnish employees: a cross-sectional study
Source: BMC Public Health. 2016 Aug 2;16:701. doi: 10.1186/s12889-016-3391-4 (PMC4971625; doi:10.1186/s12889-016-3391-4)
Supplement: Additional file 4: Figure S1. — An example (1 of the 3 days) of the feedback from the wellbeing assessment of the participants. (PDF 262 kb) [file 12889_2016_3391_MOESM4_ESM.pdf]

LIFESTYLE ASSESSMENT

Person: Ellie Example

Age	50	Activity Class	6.0 (Good)
Height (cm)	170	Resting heart rate	35
Weight (kg)	62	Max. heart rate	189
Body Mass Index	21.5		

Measurement:

Start time	Mon 01.10.2015 06:00
Duration	24h 0min
Heart rate (low/avg./high)	39 / 62 / 126

Stress reactions · Recovery · Physical activity · Daily physical activity · Heart rate · Missing heart rate 4%

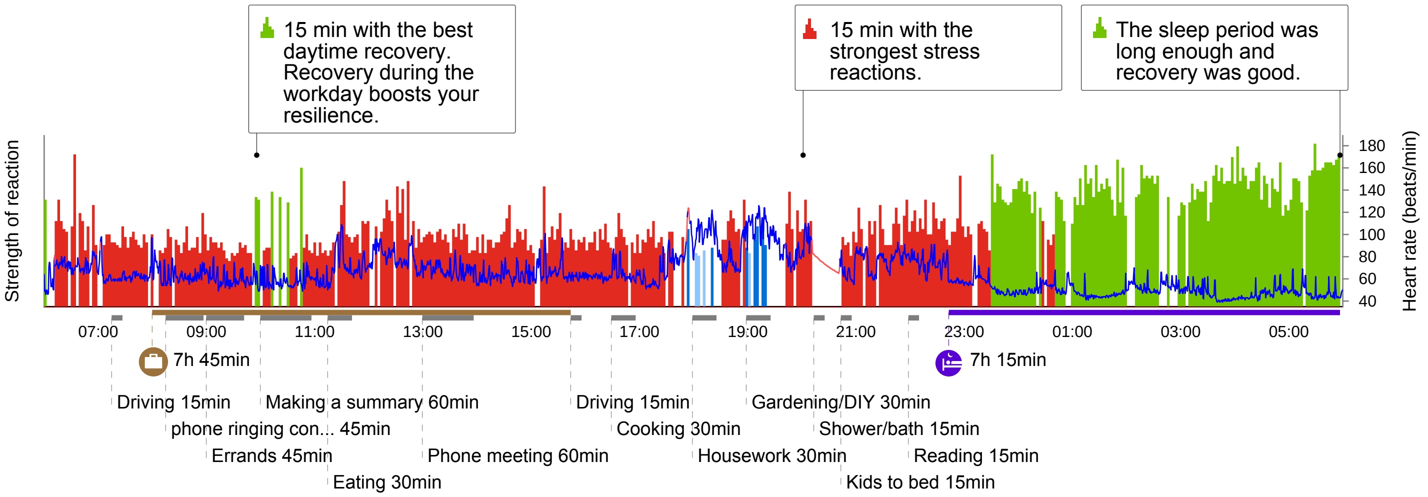

STRESS AND RECOVERY

- Stress reactions (13h 6min)
- Recovery (5h 31min)
- Physical activity (21min)
- Daily physical activity (11min)
- Other state (4h 50min)

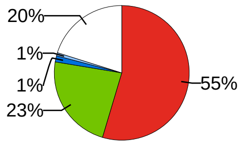

		More than usual	Normal	Less than usual
% of Stress reactions (day & night):	**55%**	> 60%	**40 - 60%**	< 40 %

		Low	Moderate	Good
% of Recovery (day & night):	**23%**	< 20%	**20 - 29%**	≥ 30%

WORK

Length of work periods: **7h 45min**

Amount of recovery during work: **11min**

Low	Moderate	Good
< 10 min	**10 - 29 min**	≥ 30 min

SLEEP

Self-reported sleep quality:

		Poor	Moderate	Good
Length of sleep:	**7h 15min**	< 5,5h	5,5h - 7h	**≥ 7h**
% of Recovery during sleep:	**72%**	< 50%	**50 - 74%**	≥ 75%
Quality of recovery (HR variability, RMSSD):	**53 ms**	0 - 15 ms	16 - 30 ms	**≥ 30 ms**

PHYSICAL ACTIVITY

The total duration of physical activity **21min** of which fitness-improving physical activity **0min.**

Physical activity index: **19/100**

Low	Moderate	Good
**0 - 29**	30 - 59	60 - 100

ENERGY EXPENDITURE

Total energy expenditure:

1604 kcal

- Physical activity 80 kcal
- Daily physical activity 109 kcal
- Other 1415 kcal
